# Supplementary material for: Impact of Clinical Pharmacy Expansion within a Rural Federally Qualified Health Center through Implementation of Pharmacist-Led Medicare Annual Wellness Visits
Source: Pharmacy (Basel). 2022 Nov 29;10(6):160. doi: 10.3390/pharmacy10060160 (PMC9781021; doi:10.3390/pharmacy10060160)
Supplement: Supplementary file 1 [file pharmacy-10-00160-s001.zip › pharmacy-2004330-supplementary.pdf]

Table S1. Screenings Identified as Due and Subsequently Ordered during Annual Wellness Visit by Providers and Pharmacists.

Interventions	Screenings Due for Patients seen by Providers; n	Screenings Ordered by Providers; n (% of due)	Screenings Due for Patients seen by Pharmacists; n	Screenings Ordered by Pharmacists; n (% of due)	Difference in the Probability of Screenings Ordered between Providers and Pharmacists; Risk Difference (95% CI)
Colon Cancer Screening	44	26 (59.1)	38	24 (63.2)	−0.041 (−0.252, 0.171)
DEXA Scan	39	21 (53.8)	46	32 (69.6)	−0.157 (−0.363, 0.048)
Mammogram	33	23 (69.7)	27	17 (63)	0.067 (−0.173, 0.308)
Pap Smear*	9	7 (77.8)	3	2 (66.7)	0.111 (−0.421, 0.717)
Lung Cancer Screening*	11	5 (45.5)	16	11 (68.8)	-0.233 (−0.588, 0.157)
Aortic Aneurysm Screening	15	8 (53.3)	12	6 (50)	0.033 (−0.346, 0.413)
Influenza Vaccine	78	43 (55.1)	39	18 (46.2)	0.090 (−0.102, 0.281)
Boostrix® Vaccine*	29	2 (6.9)	27	7 (25.9)	−0.190 (−0.408, 0.008)
Pneumonia Vaccine	42	16 (38.1)	34	17 (50)	−0.119 (−0.342, 0.104)
Shingrix™ Vaccine†	113	57 (50.4)	96	20 (20.8)	0.296 (0.173, 0.419)
COVID Vaccine†	75	9 (12)	55	33 (60)	−0.480 (−0.629, −0.331)

Chi-square tests were used to calculate the differences between screenings ordered (yes/no) for providers vs. pharmacists. If a small p-value (p-value < 0.05) for the test occurs, this indicates that the null hypothesis of equal proportions can be rejected and that the proportions are unequal. We further tested if the difference in the probability of screenings ordered between providers and pharmacists. We provide an estimate of the difference in probability of screenings ordered as well as a confidence interval. If the confidence limits do not include zero as a likely value of the population mean difference, the difference is significant at the 0.05 level. † Chi-square p-value < 0.0001; * Due to small cell sizes, Fisher Exact test was utilized and exact confidence intervals were calculated.

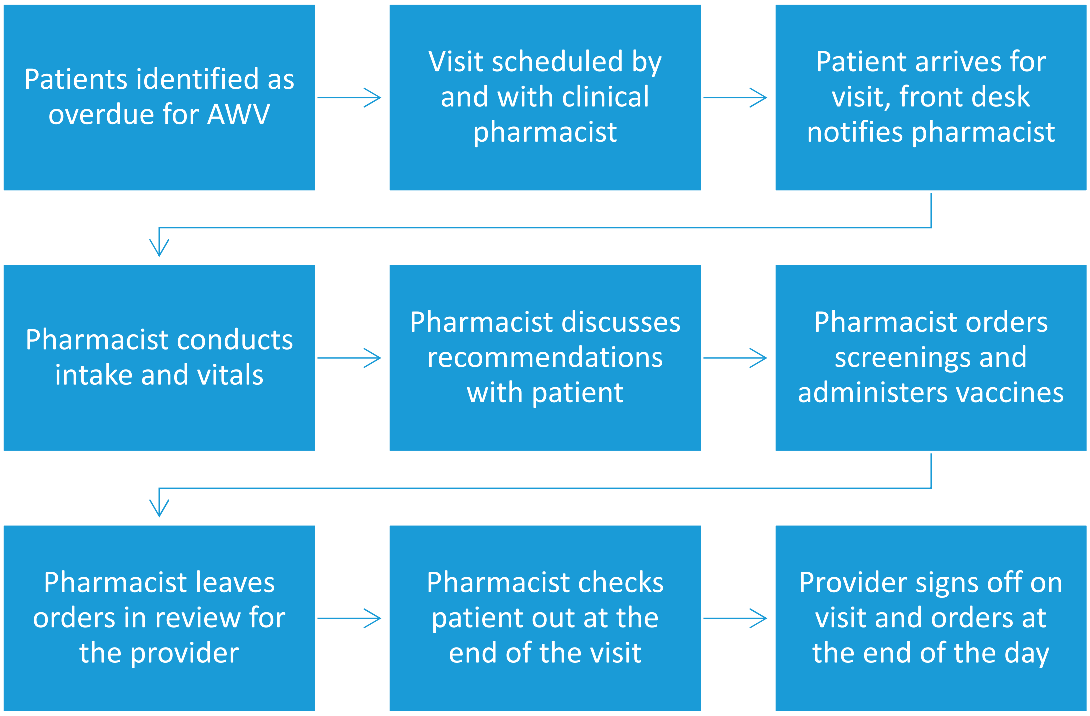

Figure S1. AWV Workflow in FQHC Outpatient Clinic.
